# Brain Distribution of Dual ABCB1/ABCG2 Substrates Is Unaltered in a Beta-Amyloidosis Mouse Model

**DOI:** 10.3390/ijms21218245

**Published:** 2020-11-03

**Authors:** Thomas Wanek, Viktoria Zoufal, Mirjam Brackhan, Markus Krohn, Severin Mairinger, Thomas Filip, Michael Sauberer, Johann Stanek, Thomas Pekar, Jens Pahnke, Oliver Langer

**Affiliations:** 1 Preclinical Molecular Imaging, AIT Austrian Institute of Technology GmbH, 2444 Seibersdorf, Austria; viktoria.zoufal@chello.at (V.Z.); Severin.Mairinger@chuv.ch (S.M.); thomas.filip@ait.ac.at (T.F.); michael.sauberer@ait.ac.at (M.S.); johann.stanek@ait.ac.at (J.S.); oliver.langer@meduniwien.ac.at (O.L.); 2 Department of Neuro-/Pathology, University of Oslo (UiO) and Oslo University Hospital (OUS), 0424 Oslo, Norway; mirjam.brackhan@medisin.uio.no (M.B.); markus.krohn@uni-luebeck.de (M.K.); jens.pahnke@medisin.uio.no (J.P.); 3 Biomedical Analytics, University of Applied Sciences Wiener Neustadt, 2700 Wiener Neustadt, Austria; thomas.pekar@fhwn.ac.at; 4 LIED, University of Lübeck, 23562 Lübeck, Germany; 5 Department of Pharmacology, Faculty of Medicine, University of Latvia, 1586 Rīga, Latvia; 6 Department of Clinical Pharmacology, Medical University of Vienna, 1090 Vienna, Austria; 7 Department of Biomedical Imaging und Image-guided Therapy, Division of Nuclear Medicine, Medical University of Vienna, 1090 Vienna, Austria

**Keywords:** ABCG2, ABCB1, blood-brain barrier, PET, Alzheimer’s disease, beta-amyloid, tariquidar, erlotinib

## Abstract

Background: ABCB1 (P-glycoprotein) and ABCG2 (breast cancer resistance protein) are co-localized at the blood-brain barrier (BBB), where they restrict the brain distribution of many different drugs. Moreover, ABCB1 and possibly ABCG2 play a role in Alzheimer’s disease (AD) by mediating the brain clearance of beta-amyloid (Aβ) across the BBB. This study aimed to compare the abundance and activity of ABCG2 in a commonly used β-amyloidosis mouse model (APP/PS1-21) with age-matched wild-type mice. Methods: The abundance of ABCG2 was assessed by semi-quantitative immunohistochemical analysis of brain slices of APP/PS1-21 and wild-type mice aged 6 months. Moreover, the brain distribution of two dual ABCB1/ABCG2 substrate radiotracers ([^11^C]tariquidar and [^11^C]erlotinib) was assessed in APP/PS1-21 and wild-type mice with positron emission tomography (PET). [^11^C]Tariquidar PET scans were performed without and with partial inhibition of ABCG2 with Ko143, while [^11^C]erlotinib PET scans were only performed under baseline conditions. Results: Immunohistochemical analysis revealed a significant reduction (by 29–37%) in the number of ABCG2-stained microvessels in the brains of APP/PS1-21 mice. Partial ABCG2 inhibition significantly increased the brain distribution of [^11^C]tariquidar in APP/PS1-21 and wild-type mice, but the brain distribution of [^11^C]tariquidar did not differ under both conditions between the two mouse strains. Similar results were obtained with [^11^C]erlotinib. Conclusions: Despite a reduction in the abundance of cerebral ABCG2 and ABCB1 in APP/PS1-21 mice, the brain distribution of two dual ABCB1/ABCG2 substrates was unaltered. Our results suggest that the brain distribution of clinically used ABCB1/ABCG2 substrate drugs may not differ between AD patients and healthy people.

## 1. Introduction

The major pathohistological hallmarks of Alzheimer’s disease (AD) are the accumulation of beta-amyloid (Aβ) plaques and neurofibrillary tangles consisting of hyperphosphorylated tau protein in the brain. It is believed that one of the underlying causes of this cerebral Aβ accumulation is the impaired clearance of Aβ peptides from the brain [1,2,3]. There are several different mechanisms for the removal of Aβ peptides from the brain [4]; one important mechanism is its transport across the blood-brain barrier (BBB) into the blood. The adenosine triphosphate-binding cassette (ABC) transporter ABCB1 (also known as P-glycoprotein), which is expressed in the luminal (blood-facing) membrane of brain capillary endothelial cells, has been shown to work together with the low-density lipoprotein receptor-related protein 1 (LRP1) in the abluminal membrane of endothelial cells in translocating Aβ peptides across the BBB [5,6,7,8,9]. There is evidence that the abundance and activity of ABCB1 are reduced in the brains of AD patients relative to age-matched healthy control subjects [10,11,12,13,14]. Studies in β-amyloidosis mouse models indicated that the activity of cerebral ABCB1 can be pharmacologically induced (e.g., by treatment with pregnane X receptor activators), leading to enhanced Aβ clearance from the brain, which may constitute a potential therapeutic target in AD [8,15,16,17,18].

At the BBB, ABCB1 is co-localized with ABCG2 (also known as breast cancer resistance protein). ABCB1 and ABCG2 have a largely overlapping substrate spectrum and act as highly efficient gate keepers in preventing the brain distribution of a range of different drugs, such as most currently known molecularly targeted anticancer drugs [19,20]. While the role of ABCB1 in mediating Aβ clearance across the BBB has been thoroughly investigated [5,6,7,8,9,21], considerably less is known with respect to ABCG2. It has been shown that ABCG2 can also transport Aβ peptides [22,23,24]. There are conflicting data regarding the abundance of ABCG2 in the brains of AD patients versus age-matched healthy controls. One study found an increase [22], another study a decrease [12] and four other studies found no changes in the abundance of ABCG2 in AD patients [10,11,25,26]. As the abundance of ABCG2 may not always correlate with its activity, it would be preferable to directly measure ABCG2 activity at the BBB of AD patients to further investigate the possible role of ABCG2 in the brain clearance of Aβ.

Positron emission tomography (PET) imaging with radiolabeled transporter substrates has been proposed as a powerful method to measure the activity of ABCB1 at the BBB [13,14]. While several effective PET tracers for ABCB1 have been described [27], ABCG2-selective PET tracers are currently not available. We have recently developed a PET protocol to measure ABCG2 activity at the mouse and human BBB [28,29,30]. This protocol is based on PET scans with the dual ABCB1/ABCG2 substrate [^11^C]tariquidar [31] under conditions of complete ABCB1 inhibition achieved by co-administration of unlabeled tariquidar [28,29,30]. In mice, the contribution of ABCG2 to the brain efflux of [^11^C]tariquidar can be revealed by administration of the ABCG2 inhibitor Ko143 [28,29].

In the present study, we first used immunohistochemistry to stain ABCG2 in the brains of a β-amyloidosis mouse model (APP/PS1-21) [32] and control mice (both aged 6 months), which revealed a significant reduction in ABCG2-stained microvessels in APP/PS1-21 mice. We then applied PET imaging to measure the consequences of the decreased abundance of cerebral ABCG2 on the brain distribution of two dual ABCB1/ABCG2 substrate radiotracers ([^11^C]tariquidar and [^11^C]erlotinib) in APP/PS1-21 mice. The brain distribution of both radiotracers did not significantly differ between APP/PS1-21 mice and wild-type mice, suggesting that the observed reduction in cerebral ABCG2 abundance may not be sufficient to alter the brain distribution of ABCB1/ABCG2 substrate drugs.

## 2. Results

### 2.1. Immunohistochemistry

**Figure 1:**
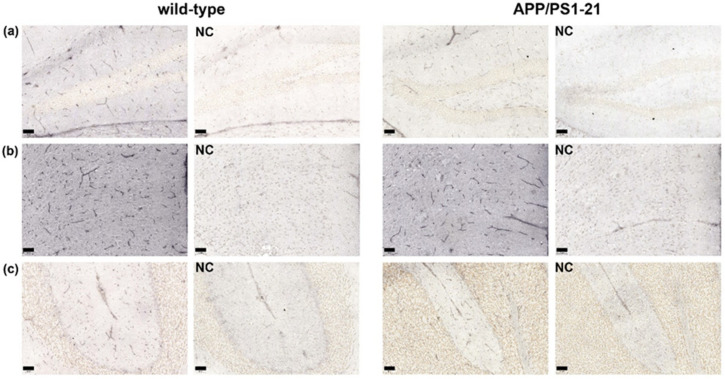
Examples of immunohistochemical staining of mouse ABCG2 in microvessels of 6-months-old wild type and APP/PS1-21 mice in (**a**) hippocampus (dentate gyrus), (**b**) cortex (cingulate cortex) and (**c**) cerebellum (4th and 5th cerebellar lobules with primary fissure) region. Enlarged areas are shown at 20× magnification. Scale bar in lower left indicates 50 µm. (NC, negative control: staining protocol without primary antibody).

**Figure 2:**
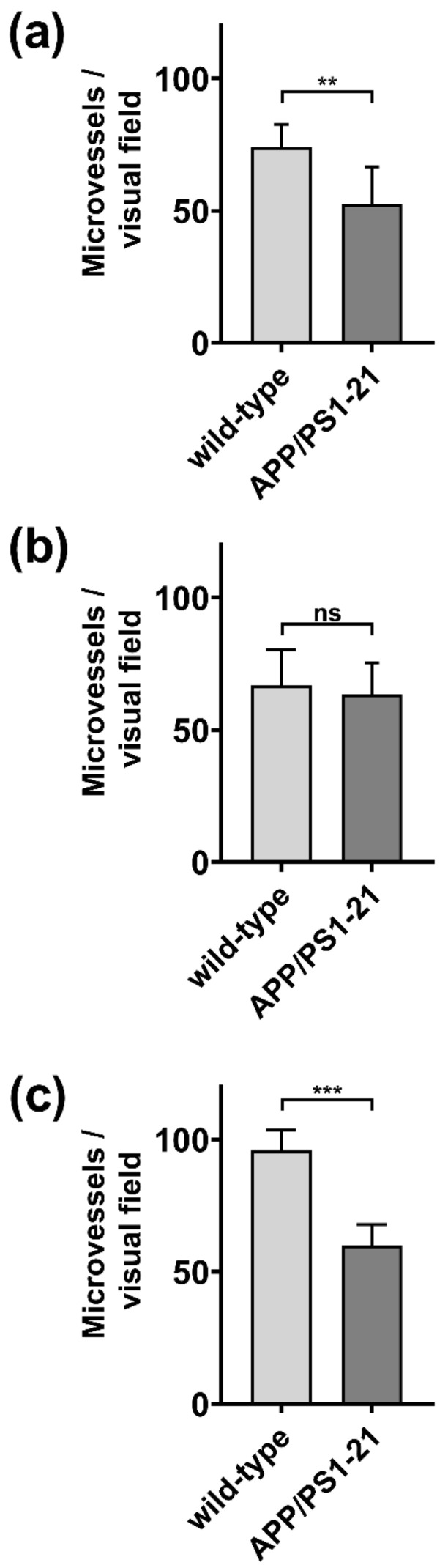
Semi-quantitative analysis of ABCG2-stained microvessels in (**a**) hippocampus, (**b**) cortex and (**c**) cerebellum of 6-months-old APP/PS1-21 mice and age-matched wild-type mice. For each region, the mean of four visual fields (at 20× digital magnification) per animal (*n* = 3 animals per strain) was used for statistical testing. Error bars indicate SD. (ns, not significant; ** *p* < 0.01; *** *p* < 0.001; 2-sided *t*-test).

ABCG2 was immunohistochemically stained in brain slices of APP/PS1-21 mice and wild-type littermates aged 6 months (*n* = 3 per group) (Figure 1). For immunohistochemical analysis, we selected two brain regions in which Aβ load was shown to be high in APP/PS1-21 mice at the investigated age (hippocampus and cortex) and one region with negligible Aβ load (cerebellum) [33]. A semi-quantitative analysis of the stained microvessels indicated a significant decrease (by 29–37%) in the number of ABCG2-stained microvessels in the hippocampus and cerebellum of APP/PS1-21 versus wild-type mice (Figure 2a,c), while no significant difference was found in the cortex (Figure 2b). This suggested that the reduction in the abundance of ABCG2 in APP/PS1-21 mice was independent of Aβ deposition.

### 2.2. [^11^C]Tariquidar PET

**Figure 3:**
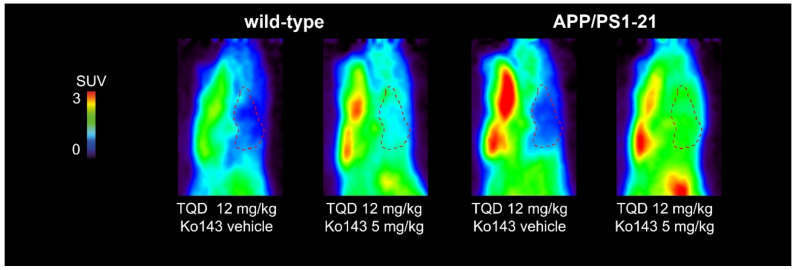
Sagittal (median axis) PET summation images (0–60 min) of 6-months-old APP/PS1-21 mice and age-matched wild-type mice pretreated i.v. with tariquidar (TQD, 12 mg/kg) at 2 h and Ko143 vehicle solution or Ko143 (5 mg/kg) at 1 h prior to [^11^C]tariquidar PET. Whole brain region is outlined with a red broken line. All images are set to the same intensity scale (0–3 standardized uptake value, SUV).

**Figure 4:**
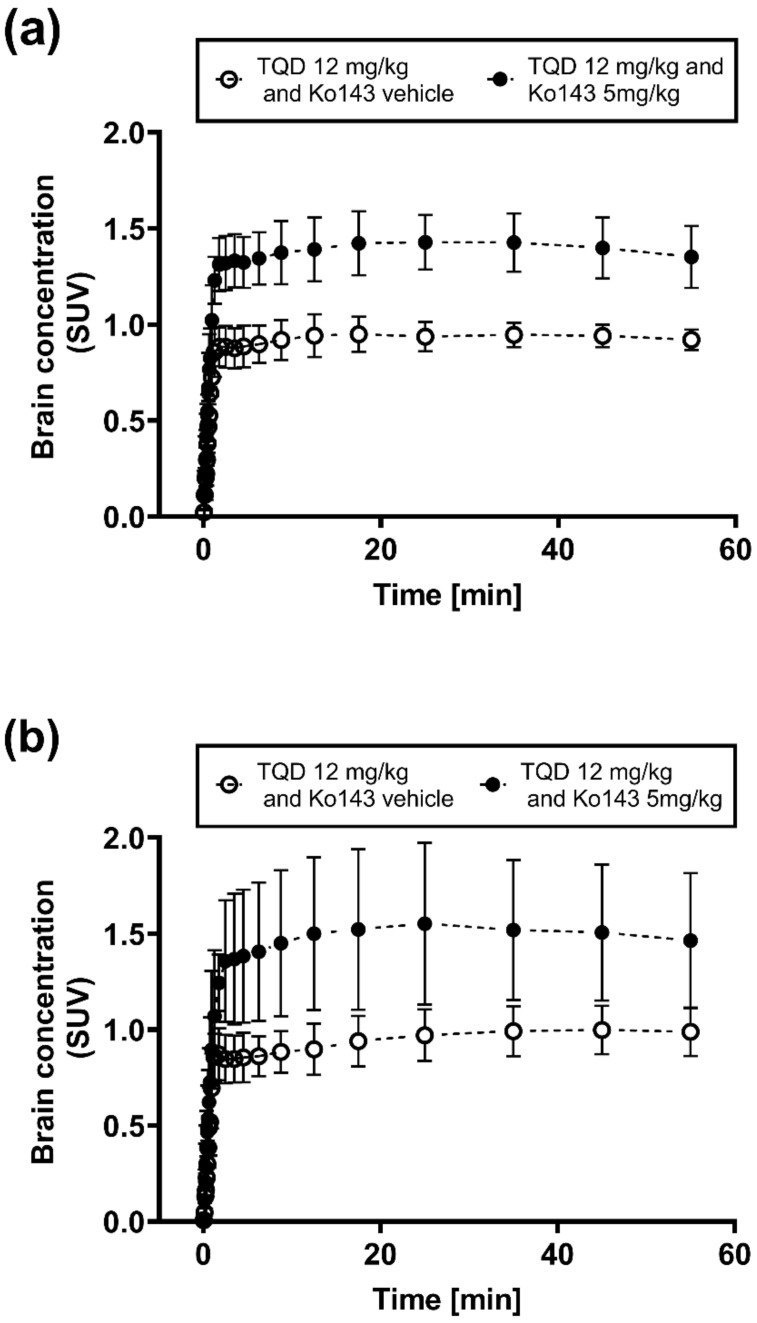
Time-activity curves (mean ± SD) of [^11^C]tariquidar in whole brains of (**a**) wild-type mice and (**b**) APP/PS1-21 mice pretreated with unlabeled tariquidar (TQD, 12 mg/kg) at 2 h and Ko143 vehicle solution (open circles, wild-type: *n* = 6, APP/PS1-21: *n* = 5) or Ko143 (5 mg/kg, closed circles, wild-type: *n* = 7, APP/PS1-21: *n* = 7) at 1 h prior to PET acquisition.

**Figure 5:**
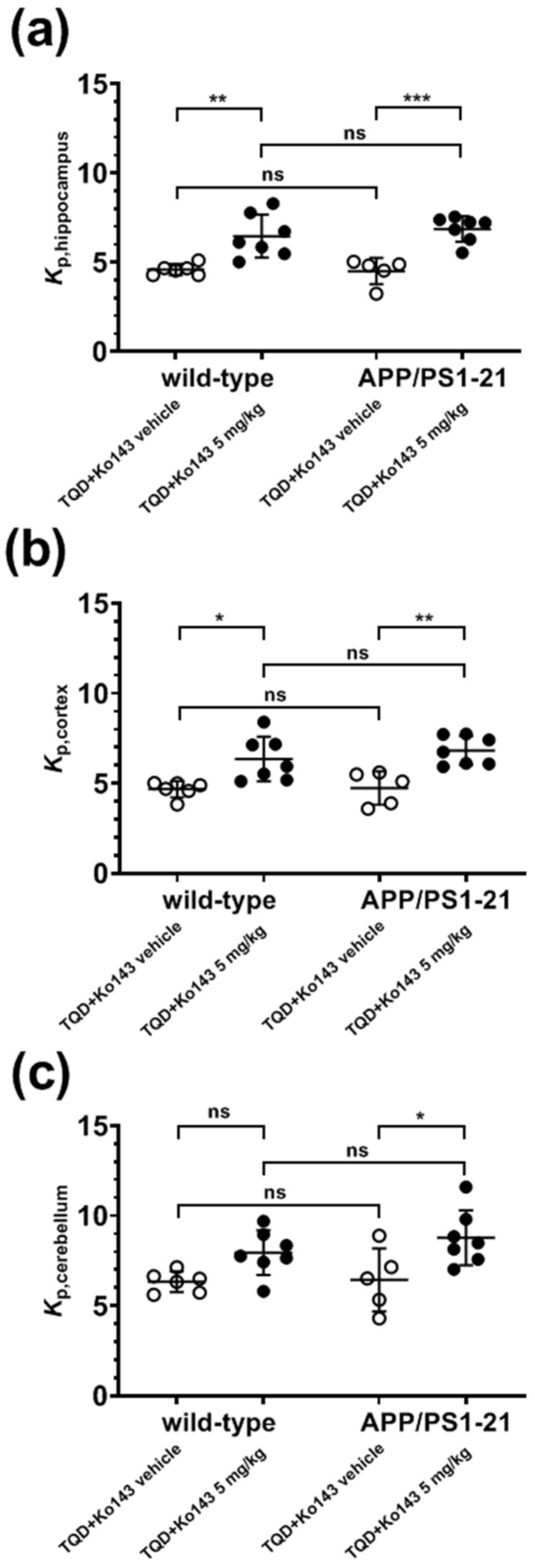
Regional brain-to-plasma radioactivity concentration ratios (*K*_p,brain_) in (**a**) hippocampus, (**b**) cortex and (**c**) cerebellum at the end of the [^11^C]tariquidar PET scan in 6-months-old APP/PS1-21 mice and age-matched wild-type mice pretreated with unlabeled tariquidar (TQD, 12 mg/kg) at 2 h and Ko143 vehicle solution (wild-type: *n* = 6, APP/PS1-21: *n* = 5) or Ko143 (5 mg/kg, wild-type: *n* = 7, APP/PS1-21: *n* = 7) at 1 h prior to the start of the PET scan. (ns, not significant; * *p* < 0.05; ** *p* < 0.01; *** *p* < 0.001; compared to Ko143 vehicle treated animals; one-way ANOVA followed by Tukey’s multiple comparison test).

We used a previously developed PET protocol [28] which involved PET scans with the dual ABCB1/ABCG2 substrate [^11^C]tariquidar with co-administration of unlabeled tariquidar (12 mg/kg) to saturate cerebral ABCB1 activity and thereby selectively measure ABCG2 activity, without and with partial ABCG2 inhibition with the ABCG2 inhibitor Ko143 (5 mg/kg) [34]. PET summation images of [^11^C]tariquidar in APP/PS1-21 and wild-type mice are shown in Figure 3. Without Ko143 pretreatment, brain radioactivity concentrations in both mouse strains were markedly lower than in most of the surrounding head region, while in Ko143-treated animals, brain radioactivity concentrations approached the concentrations in the surrounding head region. The corresponding time-activity-curves (TACs) in whole brains are shown in Figure 4. In both mouse strains, mean TACs were higher under conditions of partial ABCG2 inhibition than under conditions without ABCG2 inhibition. Brain-to-plasma radioactivity concentration ratios (*K*_p,brain_) were determined as a parameter for the brain distribution of [^11^C]tariquidar (i.e., the ratio of PET-derived radioactivity concentration at the last time point and the radioactivity concentration in plasma measured at the end of the PET scan) [28]. In Figure 5, *K*_p,brain_ values are shown for the two mouse strains for the three examined brain regions (hippocampus, cortex and cerebellum) without and with ABCG2 inhibition. No significant differences in *K*_p,brain_ values were found between APP/PS1-21 and wild-type mice in any of the investigated brain regions, either for scans without or for scans with partial ABCG2 inhibition. For both mouse strains, *K*_p,brain_ values in the hippocampus and cortex were significantly higher after partial ABCG2 inhibition (Figure 5a,b). In the cerebellum, *K*_p,brain_ was only significantly increased after partial ABCG2 inhibition in APP/PS1-21 mice but not in wild-type mice (Figure 5c). In all three brain regions, the percentage increase in *K*_p,brain_ of [^11^C]tariquidar following ABCG2 inhibition was not significantly different between APP/PS1-21 and wild-type mice (APP/PS1-21: 36–52%, wild-type: 26–41%).

### 2.3. [^11^C]Erlotinib PET

**Figure 6:**
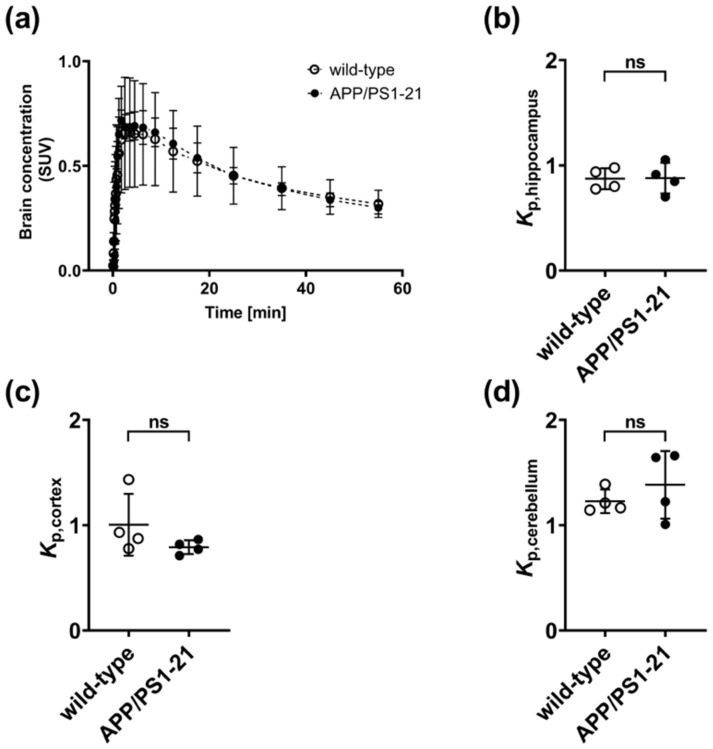
Time-activity curves (mean ± SD) of [^11^C]erlotinib in whole brains of 6-months-old APP/PS1-21 mice (closed circles, *n* = 4) and age-matched wild-type mice (open circles, *n* = 4) (**a**). Regional brain-to-plasma radioactivity concentration ratios (*K***_p,brain_**) in (**b**) hippocampus, (**c**) cortex and (**d**) cerebellum at the end of the [^11^C]erlotinib PET scan in APP/PS1-21 mice and wild-type mice (ns, not significant; 2-sided *t*-test).

To confirm the lack of an effect of the decreased abundance of ABCG2 on the brain distribution of [^11^C]tariquidar, we performed PET scans with a second ABCB1/ABCG2 substrate radiotracer ([^11^C]erlotinib) [35]. In contrast to [^11^C]tariquidar, PET scans were only performed under conditions of full ABCB1/ABCG2 activity, i.e., no ABCB1 or ABCG2 inhibitors were administered. To mimic the therapeutic usage of erlotinib, we co-injected [^11^C]erlotinib with a pharmacological dose of unlabeled erlotinib (2 mg/kg) [36]. Whole brain TACs of [^11^C]erlotinib were very similar in the two mouse strains (Figure 6a). Moreover, *K*_p,brain_ values were not significantly different between APP/PS1-21 and wild-type mice in the three examined brain regions (hippocampus, cortex and cerebellum) (Figure 6b–d).

## 3. Discussion

ABCG2 was shown to be co-localized with ABCB1 at the BBB, where both transporters limit the brain distribution of many therapeutic drugs [19,37]. Studies in transgenic mice have provided evidence for functional redundancy between ABCB1 and ABCG2 at the BBB [19,37]. In the absence of ABCB1, the transport capacity of ABCG2 usually suffices to restrict the brain distribution of dual ABCB1/ABCG2 substrates and vice versa. Only when both transporters are genetically knocked out or pharmacologically inhibited, do dual ABCB1/ABCG2 substrates show unrestricted brain distribution. Both ABCB1 and ABCG2 have been identified as Aβ transporters [5,6,7,8,9,22,23,24] and the abundance and activity of cerebral ABCB1 were found to be decreased in AD [10,11,12,13,14], which may be caused by an Aβ-induced ubiquitination, internalization and proteasomal degradation of ABCB1 [38]. It is tempting to speculate that a similar functional redundancy between ABCB1 and ABCG2 exists with respect to Aβ export as described for restricting the brain distribution of small-molecule drugs [19,37]. Xiong et al. have shown an upregulation of ABCG2 in the brains of AD patients and AD mouse models (3XTg and Tg-SwDI) by means of immunohistochemistry and Western blot [22]. Moreover, these authors used optical imaging to show that the brain concentration of fluorescent-labeled Aβ after i.v. injection was higher in *Abcg2^(−/−)^* mice as compared with wild-type mice, which confirmed Aβ transport by mouse ABCG2 [22]. Other studies, however, were not able to confirm an ABCG2 upregulation in AD brains by using either immunohistochemistry [10,11,12] or quantitative targeted proteomics [25,26].

To further address these questions, in the present study we examined both the abundance and activity of cerebral ABCG2 in a commonly employed β-amyloidosis mouse model (APP/PS1-21) [32]. This mouse model rapidly develops extensive cerebral Aβ deposits from an age of 2 months onwards [32,33] and has been used in a series of previous studies conducted in our laboratory to assess the activity of ABC transporters implicated in brain Aβ clearance with PET [15,33,39]. In a previous study we used immunohistochemical staining to show that the abundance of ABCB1 is decreased in the brains of APP/PS1-21 mice (i.e., in the hippocampus and the cortex) relative to wild-type mice of the same age range [33]. In the present study, using a comparable methodology we found a reduction in the number of ABCG2-stained microvessels in APP/PS1-21 mice (Figure 1 and Figure 2). This reduction was not only found in the hippocampus, a brain region with high Aβ load, but also in the cerebellum, in which Aβ load is negligible [33]. Our analysis was not able to differentiate whether the reduced abundance of ABCG2 in the brains of APP/PS1-21 mice was caused by a decrease in vascular density [40] or by a decrease in the density of the transporter [11]. Therefore, additional experiments with a different methodology (e.g., Western blot analysis of isolated brain microvessels) will be needed to further examine the regional differences in ABCG2 observed in the present study.

To assess the consequences of these parallel reductions in the abundance of both ABCB1 and ABCG2, utilizing PET imaging, we studied the brain distribution of two radiotracers that are dual ABCB1/ABCG2 substrates ([^11^C]tariquidar and [^11^C]erlotinib). As a first approach, we used a previously developed PET protocol dedicated to measuring cerebral ABCG2 activity [28,29,30]. This protocol uses the dual ABCB1/ABCG2 substrate [^11^C]tariquidar [31] co-administered with a pharmacological dose of unlabeled tariquidar (12 mg/kg), which leads to complete saturation of ABCB1 activity while ABCG2 remains fully active, thereby ABCG2 selectivity is achieved. The attainment of ABCG2 selectivity is enabled by the great difference in half-maximum inhibitory concentrations (IC_50_) of tariquidar for in vitro inhibition of its own transport by ABCB1 (IC_50_ = 17.1 nM) and ABCG2 (IC_50_ = 310.4 nM) [30]. To reveal the activity of ABCG2, [^11^C]tariquidar PET scans were performed without and with pretreatment with the ABCG2 inhibitor Ko143 [34] at a dose that only partially inhibits ABCG2. The employed dose of Ko143 (5 mg/kg) was selected based on a previous dose-response curve generated in *Abcb1a/b^(−/−)^* mice, which provided a half-maximum effect dose of Ko143 of 4.98 mg to enhance brain uptake of [^11^C]tariquidar [28]. We determined *K*_p,brain_ as the outcome parameter of the brain distribution of [^11^C]tariquidar, which was in a similar range in both APP/PS1-21 and wild-type mice after 5 mg/kg Ko143 (*K*_p,brain_ range: 6–8, see Figure 5) as in *Abcb1a/b^(−/−)^* mice pretreated with 5 mg/kg Ko143 [28]. Maximum brain uptake of [^11^C]tariquidar amounted to a *K*_p,brain_ of approximately 15 in *Abcb1a/b^(−/−)^* mice pretreated with 15 mg/kg Ko143, which was comparable to the brain uptake of [^11^C]tariquidar in *Abcb1a/b^(−/−)^Abcg2^(−/−)^* mice [28]. Taken together, the present data as well as the previous Ko143 dose-response data strongly suggest that the dose of Ko143 employed in our study (5 mg/kg) led to only partial ABCG2 inhibition at the mouse BBB. The experimental paradigm of studying transporter activity with a radiolabeled substrate by employing an inhibitor administered at a dose that only partially inhibits the transporter has been successfully employed by our group and by others to measure the activity of ABCB1 in the rodent and human brain with the ABCB1 substrate (*R*)-[^11^C]verapamil [33,41,42,43,44]. For instance, only PET scans after partial inhibition of ABCB1 revealed significant differences in the brain distribution of (*R*)-[^11^C]verapamil in APP/PS1-21 versus wild-type mice, while no differences were observed in baseline scans without ABCB1 inhibition [33]. In contrast to this previous study, we failed to detect differences in [^11^C]tariquidar brain distribution between APP/PS1-21 and wild-type mice, both under conditions of full ABCG2 activity and partial ABCG2 inhibition (Figure 5).

To confirm this apparent lack of difference in ABCG2 activity between APP/PS1-21 and wild-type mice, we also performed PET imaging in APP/PS1-21 and wild-type mice with a second dual ABCB1/ABCG2 substrate radiotracer. For this we used [^11^C]erlotinib, which is structurally identical to the epidermal growth factor receptor (EGFR)-targeting tyrosine kinase inhibitor erlotinib, a clinically used drug for the treatment of non-small cell lung cancer. Patients with this type of cancer often develop brain metastases, which are difficult to treat with erlotinib, most likely due to its low brain distribution caused by ABCB1/ABCG2-efflux transport at the BBB. We have previously shown that [^11^C]erlotinib is transported by mouse ABCB1 and ABCG2 and can be used to assess the functional redundancy between ABCB1 and ABCG2 at the mouse BBB [35]. To mimic the clinical use of this drug we co-injected [^11^C]erlotinib with a pharmacological dose of unlabeled erlotinib (2 mg/kg) [36]. Similar to the results obtained with [^11^C]tariquidar, we found no differences in the brain distribution of [^11^C]erlotinib between APP/PS1-21 and wild-type mice (Figure 6).

Our results are in line with and extend previous studies that failed to detect differences in the brain distribution of different drugs (including several ABCB1 substrates) between different β-amyloidosis mouse models and wild-type mice [15,45,46,47]. For instance, Gustafsson et al. found no differences in the unbound brain-plasma concentration ratios (*K*_p,uu,brain_) of the ABCB1 substrates paliperidone and digoxin between tg-APP_ArcSwe_ and age-matched control mice [46]. Similarly, Mehta et al. found no changes in the brain distribution of the ABCB1 substrates loperamide, verapamil and digoxin in triple transgenic AD mice harboring three mutant genes (*APP_swe_*, *PS-1_M146V_* and *tau_P301L_*), despite a reduction in the abundance of ABCB1 in this mouse model as shown by Western blot analysis of isolated cerebral microvessels [45]. This was explained by Mehta et al. by a thickening of the basement membrane of the BBB in AD mice, which may have impeded transcellular diffusion and thereby counteracted the reduction in the abundance of ABCB1. An alternative explanation for these previous findings may be that the changes in ABCB1 abundance in the AD mouse models were too low to cause significant changes in the brain distribution of ABCB1 substrates. ABCB1 is a high-capacity transporter, whose abundance needs to be reduced by >50% to see >2-fold changes in the brain distribution of its substrates [48]. In line with this hypothesis, we have shown that the brain distribution of the ABCB1 substrate radiotracers [^11^C]*N*-desmethyl-loperamide and (*R*)-[^11^C]verapamil was increased by only 1.1- and 1.5-fold, respectively, in heterozygous *Abcb1a/b* knockout mice (*Abcb1a/b^(+/–)^*)*,* which have a 50% reduction in the abundance of ABCB1 at the BBB, as compared with wild-type mice [49]. In contrast, in homozygous *Abcb1a/b* knockout mice (*Abcb1a/b*^(−/−)^), which completely lack ABCB1, the brain distribution of [^11^C]*N*-desmethyl-loperamide and (*R*)-[^11^C]verapamil was increased by 2.8- and 3.9- fold, respectively, relative to wild-type mice [49]. In our present study, we extended previous findings related to ABCB1 substrates [15,45,46,47] to dual ABCB1/ABCG2 substrates and showed that despite a concomitant reduction in the abundance of cerebral ABCB1 and ABCG2 in APP/PS1-21 mice as revealed by immunohistochemistry, the brain distribution of the dual ABCB1/ABCG2 substrates [^11^C]tariquidar and [^11^C]erlotinib was unaltered. While caution is warranted in extrapolating these results to humans, our results suggest that the brain distribution of clinically used ABCB1/ABCG2 substrate drugs may be unaffected by possible disease-induced alterations in transporter abundances at the BBB of AD patients.

Limitations of our study include the low number of animals used for immunohistochemical analysis, our inability to differentiate between a reduction in vascular density and a reduction in the density of ABCG2, and the lack of further experimental data to confirm the reduction in cerebral ABCG2 (e.g., Western blot analysis of ABCG2 in isolated brain microvessels).

## 4. Materials and Methods

### 4.1. General

Unless otherwise stated, all chemicals were purchased from Sigma-Aldrich Chemie (Schnelldorf, Germany) or Merck (Darmstadt, Germany) and were of analytical grade and used without further purification. The ABCB1 inhibitor tariquidar dimesylate [50] was obtained from Haoyuan Chemexpress Co. Ltd. (Shanghai, China). The ABCG2 inhibitor Ko143 [34] was purchased from Enzo Life Sciences AG (Lausen, Switzerland) or MedChemExpress LLC (Monmouth Junction, NJ, USA). Isoflurane was obtained from VIRBAC S.A. (Carros, France). Directly prior to administration, tariquidar dimesylate was freshly dissolved in 2.5% (*w*/*v*) aqueous (aq.) dextrose solution and injected intravenously (i.v.) into mice at a volume of 4 mL/kg body weight over a period of 2 min. Ko143 was freshly dissolved in ethanol and formulated for i.v. administration in a solution containing 10% (*v*/*v*) Kolliphor HS 15 and 80% (*v*/*v*) sterile aqueous saline to a final ethanol concentration of 10% (*v*/*v*). Formulated Ko143 solution was injected into mice at a volume of 2 mL/kg body weight over a period of 3–5 min.

### 4.2. Radiotracer Synthesis and Formulation

[^11^C]Tariquidar was synthesized as described previously [51]. For i.v. injection, [^11^C]tariquidar was formulated in sterile aqueous saline containing 0.01 % (*w*/*v*) Tween 80 to an approximate concentration of 370 MBq/mL. Radiochemical purity, as determined by radio-high performance liquid chromatography, was greater than 98%, and molar activity at the end of synthesis was >100 GBq/µmol.

[^11^C]Erlotinib was synthesized following established procedures [52] with a radiochemical purity of >98% and a molar activity of >100 GBq/µmol at end of synthesis. For administration into mice, [^11^C]erlotinb was formulated in 0.1 mM hydrochloric acid in sterile aqueous saline solution to yield a concentration of approximately 370 MBq/mL.

### 4.3. Animals

Female transgenic mice, which express mutated human amyloid precursor protein (APP) and presenilin 1 (PS1) under control of the Thy1-promoter (APP_KM670/671NL_, PS_L166P_) (referred to as APP/PS1-21 mice) [32,33] and age-matched wild-type littermates in a C57BL/6J genetic background were maintained at the University of Oslo and transferred to the imaging site at least three weeks prior to the PET examinations. In total, 50 mice were used in the experiments. All animals were housed in groups of 3–5 animals in individually ventilated (IVC) type III cages under controlled environmental conditions (22 ± 3 °C, 40% to 70% humidity, 12-h light/dark cycle) and had free access to standard laboratory animal diet (ssniff R/M-H, ssniff Spezialdiäten GmbH, Soest, Germany) and water ad libitum. The study was reviewed by the responsible national authorities (Amt der Niederösterreichischen Landesregierung) and approved under study numbers LF1-TVG-48/003-2014 approval date: 08 January 2015 and LF1-TVG-48/044-2019 approval date: 07 May 2019. All study procedures were in accordance with the European Community’s Council Directive of September 22, 2010 (2010/63/EU). The animal experimental data reported in this study are in compliance with the ARRIVE (Animal Research: Reporting In Vivo Experiments) guidelines.

### 4.4. Experimental Design and Pretreatment

Female APP/PS1-21 and wild-type animals were assigned to the respective groups as shown in Table 1. Two PET imaging approaches for assessing the activity of ABCG2 at the mouse BBB were performed using the two ABCB1/ABCG2 substrate radiotracers [^11^C]tariquidar [28,31] and [^11^C]erlotinib [35]:

(1) For PET imaging using [^11^C]tariquidar, groups of APP/PS1-21 and wild-type mice aged 185 ± 5 days and weighing 27.2 ± 3.0 g were pretreated two hours prior to PET with unlabeled tariquidar administered i.v. at a dose of 12 mg/kg in awake condition. Subsequently, animals were anesthetized using isoflurane/air, a catheter (Instech Lab. Inc., Plymouth Meeting, PA, USA) was introduced into a lateral tail vein and Ko143 at a dose of 5 mg/kg or Ko143 vehicle solution was additionally administered to the animals i.v. one hour prior to the start of PET acquisition. Subsequently, mice underwent a 60-min dynamic [^11^C]tariquidar PET scan. The doses of tariquidar and Ko143 were selected based on previous work to achieve full inhibition of ABCB1 [49] and partial inhibition of ABCG2 [28] at the mouse BBB.

(2) For PET imaging using [^11^C]erlotinib, no pretreatment was applied. Groups of APP/PS1-21 and wild-type mice aged 177 ± 2 days and weighing 25.1 ± 2.9 g were prepared for imaging following the procedures described below and underwent a 60-min dynamic [^11^C]erlotinib PET scan, in which a pharmacological dose of unlabeled erlotinib (2 mg/kg) was co-injected with [^11^C]erlotinib [36].

### 4.5. PET Imaging Procedure

For PET imaging, mice were pre-anesthetized in an induction chamber using isoflurane and then positioned in a prone position on a double imaging chamber (m2m Imaging Corp, Cleveland, OH, USA). Two mice were imaged simultaneously during one PET acquisition. Animals were warmed throughout the experiment and body temperature and respiratory rate were constantly monitored (SA Instruments Inc, Stony Brook, NY, USA). The level of isoflurane concentration was adjusted (range 1.5–3% in air) during the imaging procedure to achieve a constant level of anesthesia. The imaging chamber was positioned in the gantry of a microPET scanner (Focus 220, Siemens Medical Solutions, Knoxville, TN, USA) and a 10 min transmission scan using a rotating ^57^Co point source was recorded. Subsequently, 60-min dynamic emission scans (energy window 250–750 keV; timing window = 6 ns) were started with the injection of either [^11^C]tariquidar (25 ± 7 MBq) or [^11^C]erlotinib (22 ± 5 MBq) injected i.v. at a volume of 0.1 mL over a period of 120 s.

After completion of the PET scan, a blood sample (20–30 µL) was collected from the retro-orbital venous plexus, the mice were sacrificed by cervical dislocation and transcardially perfused using 10 mL phosphate-buffered saline. Blood was centrifuged (13,000× *g*, 4 °C, 4 min) to obtain plasma and whole brains were removed and processed for immunohistochemistry as described below. Aliquots of blood and plasma were transferred into pre-weighted test tubes and measured in a gamma-counter (HIDEX AMG Automatic Gamma Counter, Turku, Finland). Filled tubes were weighed to obtain tissue weight. The gamma-counter was calibrated using a series of tubes with decreasing activity of a ^11^C-solution. The measured radioactivity data were decay-corrected to the time of radiotracer injection and expressed as standardized uptake value (SUV), which is calculated as follows: (radioactivity per g (kBq/g)/injected radioactivity (kBq)) × body weight (g).

### 4.6. PET Data Analysis

The dynamic PET data were binned into 23 frames, which increased incrementally in time length. PET images were reconstructed using Fourier re-binning of the 3-dimensional sinograms followed by a 2-dimensional filtered back-projection with a ramp filter giving a voxel size of (0.4 × 0.4 × 0.796) mm³. Using Amide (version 1.0.4.) [53] or PMOD software (version 3.6, PMOD Technologies Ltd., Zurich, Switzerland) volumes of interest (VOI) covering the whole brain as well as the cortex, hippocampus and cerebellum region were outlined on the PET images with the aid of the Mirrione Mouse Atlas and guided by representative magnetic resonance (MR) images obtained in comparable animals on a 1 Tesla benchtop MR scanner (ICON, Bruker BioSpin GmbH, Ettlingen, Germany). From the VOIs, time-activity curves (TACs) were derived and expressed in SUV units. Whole brain and regional brain uptake was expressed as the brain-to-plasma radioactivity concentration ratio in the last PET frame (*K*_p,brain_) [28]. For calculation of *K*_p,brain_, the radioactivity concentration derived from the last PET time frame (from 50–60 min after injection) was divided by the radioactivity concentration measured with the gamma-counter in the plasma sample obtained after the PET scan.

### 4.7. Immunohistochemistry

Freshly harvested brains from animals that had undergone [^11^C]erlotinib PET scans were immediately washed with 30% aq. sucrose solution and embedded in freezing medium (Tissue-Tek, Sakura Finetek, Staufen, Germany). Samples were snap frozen in liquid nitrogen and stored at –80 °C until further processed.

After defrosting from –80 °C to –20 °C brains were cut in transversal orientation in 10 µm thick slices using a cryostat (Microm HM 550, Walldorf, Germany). Frozen sections of three brain regions including the hippocampus, cortex, and cerebellum were mounted on coated slides (VWR Superfrost Plus) and stored at –80 °C until the staining procedure was initiated.

For immunohistochemical staining of mouse ABCG2, the thawed brain slices were fixed with methanol/acetone (1:1) for 10 min at 4 °C. After washing with 0.1 M tris-buffered saline solution (TBS), endogenous peroxidase activity was quenched by incubation with 0.5% (*v*/*v*) hydrogen peroxide in TBS for 30 min. In order to obtain standardized staining results, the slides were washed and inserted into cover plates (Thermo Scientific™ Shandon™ Glass Coverplates, Fisher Scientific, Vienna, Austria). Blocking solution was added for 1 h at room temperature to suppress non-specific reactions. Anti-BCRP/ABCG2 antibody (1:400, [BXP-53, ab24115], Abcam) or antibody carrier solution (negative control) was used to incubate the brain slices overnight at 4 °C. After washing the slides with TBS, slides were incubated with the secondary antibody (1:500, Biotin-SP (long spacer) AffiniPure Donkey Anti-Rat IgG (H + L), Jackson Immuno Research, West Grove, PA, USA) for 60 min at room temperature. Following three further washing steps, the antibody signals were amplified for 60 min at room temperature employing the VectaStain ABC-Kit (Vector Laboratories Inc, Burlingame, CA, USA). After rinsing, the slides were incubated in nickel/diaminobenzidine solution for 10 min to visualize ABCG2. The slides were then washed, dehydrated and mounted using Entellan^®^ (Merck Darmstadt, Germany).

The mounted slides were scanned at (0.11 × 0.11) µm/pixel resolution using a digital slide scanner (Pannoramic Desk, 3dHistech Ltd., Budapest, Hungary) and digital images of comparable regions in the hippocampus, cortex and cerebellum of APP/PS1-21 and wild-type mice were extracted. For the semi-quantitative evaluation of stained microvessels, four visual fields (20× digital magnification) in the same brain region per mouse (*n* = 3 animals per group) were counted manually on the digital images by the same operator. The operator was not blinded to the study groups. However, the digital images were selected randomly for manual counting to avoid bias. Due to high background staining automatic analysis was not possible.

### 4.8. Statistical Analysis

To analyze differences between two groups a 2-sided *t*-test and between multiple groups a one-way ANOVA followed by a Tukey’s multiple comparison test were employed using Prism 8 software (GraphPad Software, La Jolla, CA, USA). The level of statistical significance was set to *p* < 0.05. All values are given as mean ± standard deviation (SD).

## 5. Conclusions

Despite significant reductions in the abundance of the Aβ transporters ABCB1 and ABCG2 in the brains of APP/PS1-21 mice, the brain distribution of the dual ABCB1/ABCG2 substrates [^11^C]tariquidar and [^11^C]erlotinib was unaltered relative to wild-type mice. This may be related to the high transport capacities of ABCB1 and ABCG2 and is in line and extends previous studies, which failed to detect differences in the brain distribution of diverse ABCB1 substrates between different AD mouse models and wild-type mice. While caution is warranted in extrapolating our results to the human BBB, our findings suggest that while disease-induced alterations in the abundance of ABCB1 and ABCG2 may be sufficient to decrease the brain clearance of Aβ peptides, they may not be sufficient to cause large changes in the brain distribution of clinically used ABCB1/ABCG2 substrate drugs.

## Figures and Tables

**Table 1:** Overview of examined animal groups and numbers.

**[^11^C]Tariquidar Study**		**Animal Group**	
**Pretreatment**		**Wild-Type**	**APP/PS1-21**
Tariquidar (12 mg/kg) and Ko143 vehicle	*n*	10 (4) ^1^	7 (2)
	Age [days] ^2^	189 ± 4	188 ± 1
	Body weight [g] ^2^	26.7 ± 2.1	27.9 ± 3.2
	Injected activity [MBq] ^3^	28 ± 6	25 ± 3
Tariquidar (12 mg/kg) and Ko143 (5 mg/kg)	*n*	11 (4)	12 (5)
	Age [days]	181 ± 5	183 ± 6
	Body weight [g]	28.9 ± 3.3	25.3 ± 2.4
	Injected activity [MBq]	24 ± 7	23 ± 10
**[^11^C]Erlotinib Study**		**Animal Group**	
		**Wild-Type**	**APP/PS1-21**
	*n*	5 (1)	5 (1)
	Age [days]	176 ± 1	179 ± 2
	Body weight [g]	25.1 ± 1.2	25.2 ± 4.3
	Injected activity [MBq]	19 ± 3	26 ± 4

^1^ Number in parentheses indicates drop-outs due to death/intolerability of the protocol; ^2^ At date of PET examination; ^3^ Injected amounts of radioactivity.

## Abbreviations

ABC	Adenosine triphopshate-binding cassette
ABCB1	ABC subfamily B member 1, also known as P-glycoprotein (P-gp)
ABCG2	ABC subfamily G member 2, also known as breast cancer resistance protein (BCRP)
AD	Alzheimer’s disease
Aβ	Beta-amyloid
BBB	Blood-brain barrier
*K*_p,brain_	Brain-to-plasma radioactivity concentration ratio
PET	Positron Emission Tomography
SUV	Standardized uptake value
TAC	Time-activity curve
